# Experimental Demonstration of Remote and Compact Imaging Spectrometer Based on Mobile Devices

**DOI:** 10.3390/s18071989

**Published:** 2018-06-21

**Authors:** Jie Chen, Fuhong Cai, Rongxiao He, Sailing He

**Affiliations:** 1 Mechanical and Electrical Engineering College, Hainan University, Haikou 570228, China; chenjie9294@yeah.net; 2 Institute of Tropical Agriculture and Forestry, Hainan University, Haikou 570228, China; herongxiao@gmail.com; 3 National Engineering Research Center of Optical Instrumentation, Centre for Optical and Electromagnetic Research, Zhejiang University, Hangzhou 310058, China

**Keywords:** spectroscopy, remote sensing and sensors, imaging spectrometer, handheld

## Abstract

Imaging spectrometers show great potential for environmental and biomedical sensing applications. Selfie sticks, which are tools used to take photographs or videos, have gained global popularity in recent years. Few people have connected these two objects, and few people have researched the application of imaging spectrometers to perform scientific monitoring in point-of-use scenarios. In this paper, we develop a compact imaging spectrometer (35 g in weight, 18 mm in diameter, and 72 mm in length) that can be equipped on a motorized selfie stick to perform remote sensing. We applied this system to perform environmental and facial remote sensing via motorized scanning. The absorption of chlorophyll and hemoglobin can be found in the reflectance spectra, indicating that our system can be used in urban greening monitoring and point-of-care testing. In addition, this compact imaging spectrometer was also easily attached to an underwater dome port and a quad-rotor unmanned aerial vehicle to perform underwater and airborne spectral detection. Our system offers a route toward mobile imaging spectrometers used in daily life.

## 1. Introduction

Recently, there has been a growing variety of applications that take advantage of the digital cameras in mobile devices. Photos and videos have become important parts of not only scientific work, but also daily life. There is a wealth of imaging information in photos and videos. Right now, entering an era of artificial intelligence, excellent computing systems showing advanced capabilities in processing spatial data have been developed. As a result, increasing amounts of data can be mined to reveal deep-level information. We can utilize a digital camera and smartphone apps for face and plant recognition. One straightforward idea to increase the information in these images is to upgrade a digital camera to an imaging spectrometer [1,2,3]. In this way, we can obtain a hyperspectral data cube, consisting of two spatial- and one spectral-dimension data [4,5]. Based on spectral information, many applications can be carried out, including soil classification, gas detection, food-quality control, etc. [6,7,8].

It has been demonstrated recently that incorporation of advanced optical systems into smartphone platforms is a useful development [9,10,11,12,13,14,15,16]. In our previous work, we developed a digital camera-based portable imaging spectrometer to detect reflectance spectra from the ocean, a lake, and a human hand [2,3]. The imaging spectrometer of our previous work was about 99 g in weight. Compared with scientific instruments, it was lightweight. However, this judgment is somewhat inaccurate for some applications. When we attached this imaging spectrometer to the digital cameras in more advanced consumer electronics products, e.g., a selfie stick or a quad-rotor unmanned aerial vehicle (UAV), their camera systems may not work due to the heavy weight of the imaging spectrometer. In this paper, we further reduce the weight of the imaging spectrometer to 35 g and succeed in combining this new compact imaging spectrometer with selfie stick, underwater dome port, and UAV to perform remote spectral sensing experiments. The spectral resolution is about 15 nm, and the cost of the imaging spectrometer is relatively low. These experiments demonstrate that we can acquire accurate spectral images in daily life. These results show that remote-sensing work can be achieved without the help of scientific grade instruments in the future. Furthermore, with the advantages of being low-cost and compact, our imaging spectrometers can be installed on a group of unmanned aerial vehicles or distributed in a monitoring system for sensing over a large space range.

## 2. Materials and Methods

**Figure 1:**
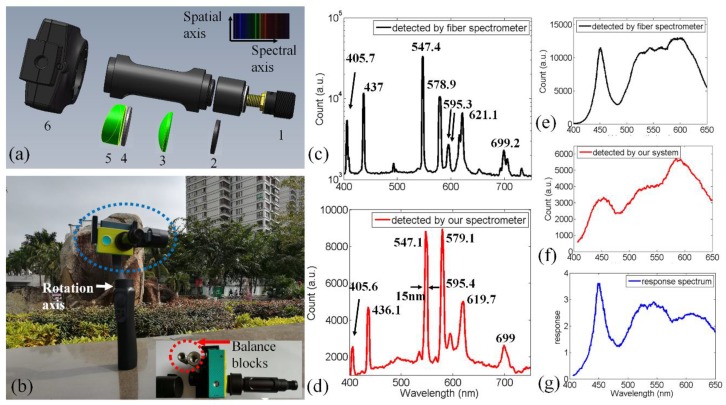
(**a**) Schematic illustration of the compact imaging spectrometer, which includes the following optical elements: imaging lens (1); slit (2); doublet lens (3); grating (4); prism (5); CMOS (6). The weight of the imaging spectrometer (without the camera sensor) is about 35 g, the diameter is 18 mm, and the length is 72 mm. A Wi-Fi CMOS camera with a 20 mm closed-circuit television (CCTV) lens is utilized to capture the spectral image. (Inset) one spectral image captured by the Wi-Fi camera when measuring a mercury lamp. (**b**) The compact imaging spectrometer can be held by a motorized selfie stick to scan objects of interest. (Inset) balance blocks are installed at the back of the selfie stick to maintain the balance of the system. (**c**) Spectrum of a mercury lamp detected by a commercial fiber spectrometer. (**d**) Spectrum of the mercury lamp detected by our imaging spectrometer. (**e**) Spectrum of a light-emitting diode (LED) lamp detected by a commercial fiber spectrometer. (**f**) Spectrum of a LED lamp detected by our imaging spectrometer. (**g**) The optical intensity response.

Figure 1a shows the system configuration. It includes an imaging lens, slit, doublet lens, grating, and prism. All of the optical elements, whose diameters are 12.7 mm, are installed in two aluminum tubes (SM05L20C and SM05L03, Thorlabs, Newton, NJ, USA). In this paper, the focal length of the imaging lens is about 20 mm, the width of the slit is 50 μm, the focal length of the doublet lens is 30 mm, the groove density of the grating is 300 grooves/mm, and the deflection angle of the prism is 10 degree. The slit is placed at the imaging plane of the imaging lens, which coincides with the front focal plane of the doublet lens. The prism is used to deflect the 1st diffraction pattern into the Complementary Metal Oxide Semiconductor (CMOS) sensor of a Wi-Fi camera [17]. The weight of the imaging spectrometer is about 35 g (without the Wi-Fi camera). The diameter is 18 mm and the length is 72 mm. The reflected light from one vertical line region of the object was captured by the imaging lens and passed through the slit. The doublet lens was used to collimate the light, which was then diffracted by the grating. After the grating and prism, the 1st-order diffraction pattern was captured by the Wi-Fi camera as a spectral image.

This imaging spectrometer consists of inexpensive off-the-shelf optical components, and the utilization of a prism-grating module ensures good imaging quality and a compact size. As shown in Figure 1a, apart from a prism, other optical elements have regular shapes and can be installed in the aluminum tubes. These regular optical elements can be mounted by two retaining rings (SM05RR, Thorlabs, Newton, NJ, USA). In our work, we used a slotted tube SM05L20C to install a doublet lens, grating and prism. For the prism, we made a plastic hollow ring through 3D printing. This plastic hollow ring is not shown in Figure 1. The shape of a plastic hollow ring is the same as that of the prism. The plastic hollow ring can provide a flat, uninterrupted mounting surface between the prism and retaining ring. The detail of the utilization of plastic hollow ring can be found in the web page of the “Round Wedge Prisms” on the Thorlabs web site. The grating was installed before the prism. Because there was a slot on the tube, we could rotate the grating to adjust the groove direction via fingers. The groove direction of the grating should be perpendicular to the edge direction of the prism. The doublet lens was installed before the grating. The doublet lens is used to collimate the light penetrating the slit. Because the slit direction should be perpendicular to the groove direction, we installed the slit in another tube (SM05L03, Thorlabs, Newton, NJ, USA). These two tubes could be connected via a mechanical thread. We could rotate the SM05L03 to adjust the slit direction. A closed-circuit television imaging lens, whose diameter is about 12 mm, was selected to capture the external light. This imaging lens was installed at the front end of the SM05L03. 

As shown in Figure 1b, a portable motorized selfie stick and a Wi-Fi camera are used as the push-broom scanning and detection module. The selfie stick and Wi-Fi camera can be wirelessly controlled by smartphone apps via Wi-Fi and Bluetooth signals. The selfie stick is designed to stabilize the Wi-Fi camera. However, as we attached the imaging spectrometer to the camera, the system became unbalanced. To maintain the stability of the system, we added balance blocks (64 g) to the back of the selfie stick to balance the additional torque generated by the imaging spectrometer, as shown in the inset of Figure 1b. It is worth mentioning that a larger focal length doublet lens could improve the system spectral resolution, but this would lead to a larger length of the imaging spectrometer, increasing the total weight and adjustment effort of the balance blocks required and the workload of the selfie stick. Our selfie stick-based imaging spectrometer is a standalone instrument that facilitates easy spectral image scanning. It only requires one person to control the system using smartphone apps. The scanning speed of the selfie stick is 1.0 degree/s, and its maximum load weight and rotating angle are 200 g and 60 degrees, respectively. In Figure 1b, the rotation axis is indicated by a white arrow and the rotation part (i.e., imaging spectrometer) is indicated by a dotted blue circle. The settings of the camera are 1920 × 1080 pixels at 25 fps and automatic ISO. The camera utilized in this paper only works on automatic ISO mode, so we selected an object with slight variations in optical intensity to perform push-broom scanning. In the future, we will upgrade our system with a better camera, whose ISO parameter and exposure time can be fixed.

Wavelength calibration is also needed before remote sensing experiments. A mercury lamp was applied as the standard optical source [18], whose spectrum was detected by a commercial fiber spectrometer (STS-VIS, OceanOptics, Oxford, UK) and is shown in Figure 1c. We used our system to capture a spectral image of the lamp, and the spectrogram is shown as an inset in Figure 1a, whose horizontal and vertical axis represent the spectral and spatial axes, respectively. After wavelength calibration, which is described elsewhere [2], each pixel index along the horizontal axis could be transferred to a wavelength number, and the digital gray value of each red-green-blue (RGB_pixel indicates the optical intensity at the corresponding spectrum. These spectral data can be considered as digital number (DN) spectra. For convenience, we refer to DN spectra as spectra in the following section. Hence, one horizontal line of the spectral image can be transformed into spectral data, as shown in Figure 1d. As shown in Figure 1c, the peak wavelengths of the spectrum detected by a commercial fiber spectrometer are 405.7 nm, 437 nm, 547.4 nm, 578.9 nm, 595.3 nm, 621.1 nm and 699.2 nm, respectively. Similarly, the peak wavelengths of the spectrum detected by our system are 405.6 nm, 436.1 nm, 547.1 nm, 579.7 nm, 595.4 nm, 619.7 nm and 699 nm, respectively, as shown in Figure 1d. The minimum, median and maximal wavelength errors are 0.1 nm, 0.3 nm and 0.9 nm, respectively. Therefore, the detected spectrum obtained by our system agrees well with that obtained by a commercial fiber spectrometer.

We also used the commercial fiber spectrometer and our imaging spectrometer to detect a light-emitting diode (LED) lamp. The results are shown in Figure 1e,f respectively. The profile of spectrum in Figure 1e differed from the profile of spectra in Figure 1f. One reason is the optical intensity response spectrum of the CMOS sensor is altered by the color filer before the sensor. We can calculate the optical intensity response spectrum (400–650 nm) of our imaging spectrometer based on these two spectra. Defining the accurate spectrum derived from the well-calibrated commercial fiber spectrometer as I_std_(λ), and the spectrum obtained by our system as I_det_(λ), then the optical intensity response can be calculated by I_std_(λ)/I_det_(λ), the result is shown in Figure 1g. In the future, we can also calculate the optical intensity response of 650 nm–1100 nm light by detecting an appropriate near-infrared light source, e.g., halogen lamp. This response spectrum can be used to calibrate the data obtained by our system.

## 3. Results and Discussion

### 3.1. Spectral Imaging for Environment Sensing

**Figure 2:**
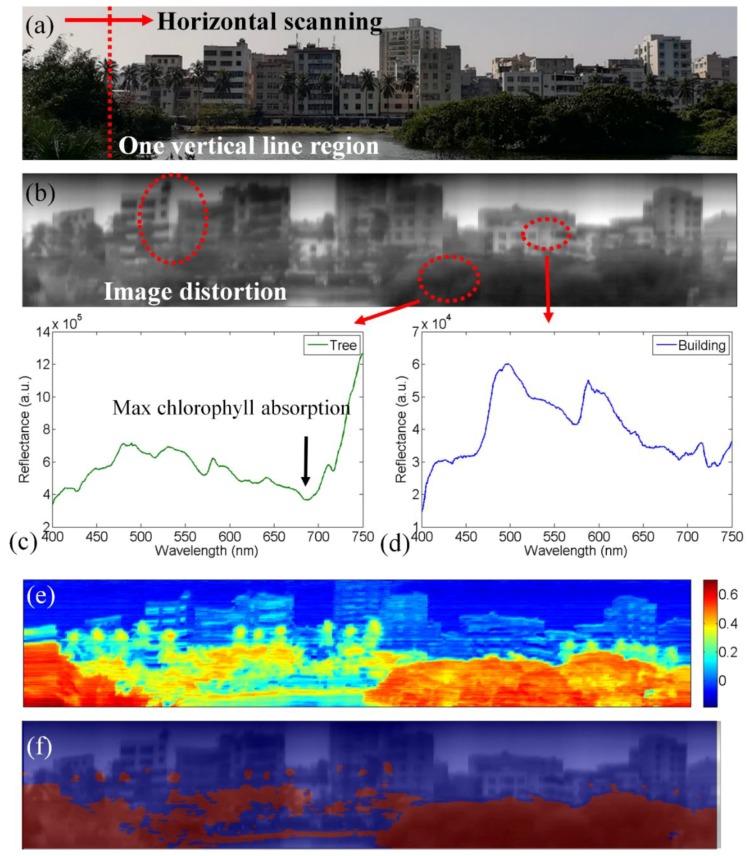
The selfie stick-based imaging spectrometer was utilized to scan environmental objects on campus. (**a**) Photo of the scanned object. The scanning was performed in the horizontal direction; (**b**) the spatial image obtained from the scanning result; (**c**) the reflectance spectrum from the tree region; (**d**) the reflectance spectrum from the building region; (**e**) the reflected light ratio index (RLRI) image based on the hyperspectral data cube; (**f**) the overlap of the spatial image and the binary image based on RLRI value.

We first used our system to perform environmental remote sensing. Traditional remote sensing is based on satellite or UAV platforms. However, aeronautical remote sensing can only give the spectral imaging data of the Earth’s surface. The spectral images of objects under a tree or building cannot be acquired. Using our selfie stick-based imaging spectrometer, we can capture spectral images from the horizontal angle of view. Various environmental objects on campus were selected as the first scanned objects. The photo of this scanned object is shown in Figure 2a. The scanning of the selfie stick and the video recording of the Wi-Fi camera were started at the same time. The slit of the imaging spectrometer was perpendicular to the scanning direction.

During horizontal scanning, a series of spectral images were stored in the recorded video. Therefore, each frame of the video contained the spectral and spatial information of one vertical line region of the scanned object. Integrating the digital gray value of all spectra bands (about 900 bands) along the spectral axis (400–750 nm), we could obtain an optical intensity of one spatial spot. After the scanning experiment, we extracted the spatial information from each fame and obtained a series of vertical line profiles. Stitching all of the vertical line profiles, a spatial image could be obtained, as shown in Figure 2b. Sometimes, the wind might shake the selfie stick, resulting in image distortion. In addition to spatial information, spectra of each pixel in the spatial image were also obtained from the scanning result. The spectra of the tree region and building region are shown in Figure 2c,d, respectively. We could find a rapid change in reflectance in the near infrared range in Figure 2c, which is the so-called ‘red edge’ [19]. The red edge effect is used to monitor plant activity. Because the normalized differential vegetation index [NDVI = (NIR − RED)/(NIR + RED)] is defined with the NIR band at 800 nm, and our system only reaches 750 nm band. Therefore we cannot utilize NDVI in this work. We define a similar ‘reflected light ratio index’ [RLRI = (NIR − RED)/(NIR + RED)], whose NIR index denotes the integral reflectance between 740 nm and 750 nm, and RED index denotes the integral reflectance between 680 nm to 690 nm. Because a leaf can absorb the 680–690 nm light and reflect 740–750 nm light, this index should have a relatively larger value at vegetation region. The results of RLRI image are shown in Figure 2e. We selected 0.3 as a threshold value to generate a binary image according to the RLRI value. If the RLRI value of one pixel is larger (smaller) than 0.3, this pixel is set as red (blue) color. Figure 2f shows the overlap of the spatial image and the binary image based on the RLRI value. The red region coincides with the vegetation region in the image. Based on binary image of RLRI value, we can also obtain the spatial image’s vegetation coverage, which is 33.76% in this work. Hence, the selfie stick-based imaging spectrometer can be useful in e.g., urban greening monitoring and forest leaf area index detection.

### 3.2. Spectral Imaging for Human Face

In the biomedical field, imaging spectrometers can be used for oxygenation and perfusion monitoring [20]. However, this kind of detection is usually limited to labs or hospitals. Acquiring spectral image data from human skin is important for health monitoring. Herein, we demonstrate the feasibility of our system for human skin imaging.

**Figure 3:**
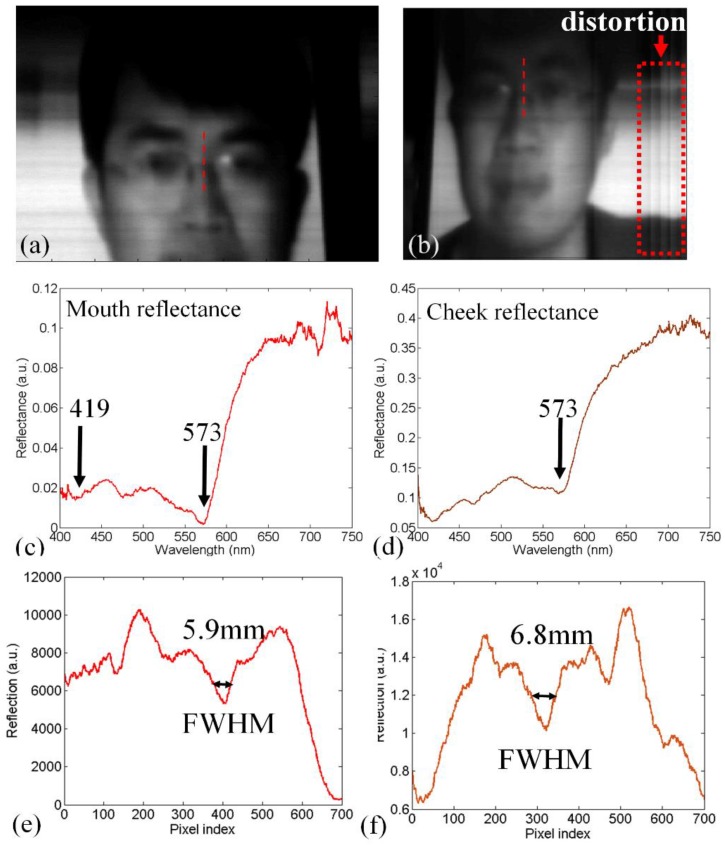
The selfie stick-based imaging spectrometer was utilized to scan the face of the second author. (**a**) The scanning results when the selfie stick was placed on a stable table; (**b**) the scanning results when the selfie stick was held by an assistant; (**c**) the reflectance spectrum from the mouth region; (**d**) the reflectance spectrum from the cheek region. (**e**,**f**) are the optical intensity profiles of the line regions indicated by the red dotted lines in (**a**,**b**), respectively.

During the scanning experiment, the selfie stick was placed on a stable table to scan the face of the second author, who controlled the system by himself using a smartphone. The distance between the face and imaging spectrometer is about 65 cm. An 11-s video was acquired. Based on the video, the spatial image was reconstructed and shown in Figure 3a. This experiment was repeated with an assistant holding the selfie stick. The distortion in the right part of Figure 3b was due to his hand shaking. It is hard to keep the hand motionless during scanning. Results indicated that the operation of our system requires a stable platform. The spectrum of the lips and the cheek could also be acquired from the scanning video, as shown in Figure 3c,d, respectively. Observing the spectrum in Figure 3c, we could find two absorption bands (419 nm and 573 nm) due to the presence of HbO_2_ (oxyhemoglobin). In contrast, since the blood concentration of the cheek is lower than that of the lips, the cheek absorbed less light at 573 nm, as shown in Figure 3d. Therefore, our system may have potential for on-site oxygenation and perfusion monitoring, which is important for the treatment of damaged skin.

In addition, we could quantitatively demonstrate the image quality based on the spatial images. We selected two line regions, indicated by two red dotted lines in Figure 3a,b. These two lines were perpendicular to a black spectacle frame on the nose. The thickness of the black spectacle frame is 1 mm. The optical intensity profiles of these two line regions are shown in Figure 3e,f. The reflection of the black spectacle frame should be less than the reflection of skin. In Figure 3e,f, we could find two corresponding local minimums, which were derived from the reflection of the black spectacle frame. As shown in Figure 3e,f, the full width at half maxima (FWHM) was 5.9 mm and 6.8 mm. We can use the FWHM index to describe the spatial resolution of our system. Therefore, when the detection distance is 65 cm, the spatial resolution is about 5.9 mm.

### 3.3. Underwater Spectral Detection for Fruit Ripeness

**Figure 4:**
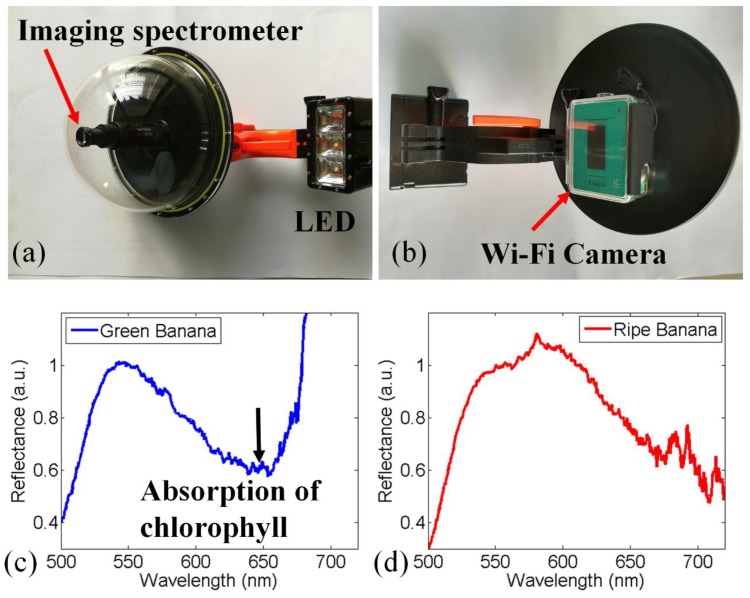
The compact imaging spectrometer can be installed in a waterproof dome port for underwater detection. (**a**,**b**) show the front and back views of the underwater spectral detection system, respectively. We sank this system into a water tank to measure the reflectance from bananas. (**c**,**d**) show the reflectance spectra from green and ripe bananas, respectively.

Due to the small size of our imaging spectrometer, it can be easily sealed in a waterproof dome port, an underwater camera attachment, as shown in Figure 4a. The diameter and weight of the waterproof dome port is 150 mm and 245 g respectively, and the waterproof depth is about 6 m. The front and back views are shown in Figure 4a,b, respectively. The imaging spectrometer can be installed at the center of the dome port with the camera placed at the back of the dome port. A white-light LED is installed next to the dome port. The dome port and LED module are designed for divers to carry out underwater photography. Therefore, this dome port-based imaging spectrometer can be used to perform underwater detection.

In our experiment, we sank this system to a depth of 0.5 m in a water tank to demonstrate its ability. Bananas were affixed to the bottom of the tank. The distance between the bananas and imaging spectrometer was about 0.2 m. The experiment was performed in a darkroom, and only LED light illuminated the bananas. The spectra from the green bananas and ripe bananas are shown in Figure 4c,d, respectively. These two spectra were normalized at 550 nm. Compared with the spectrum from the ripe bananas, the green bananas absorbed more light near the 650 nm band due to a greater amount of chlorophyll present in green bananas. Consequently, a reflectance minimum due to the absorption of chlorophyll could be found in Figure 4c. When the banana was ripe, its skin turned into yellow and almost all the chlorophyll was decomposed. The loss of chlorophyll resulted in a different reflection spectrum in the ripe banana, as shown in Figure 4d. In ripe banana skin, the main element is carotenoid, which absorbs much less light at the 650 nm band. Hence, there was no absorption peak around 650 nm in Figure 4d. This experiment demonstrates that the dome port-based imaging spectrometer can detect underwater chlorophyll.

### 3.4. Prototype of Airborne Imaging Spectrometer based on Consumer Unmanned Aerial Vehicle (UAV)

**Figure 5:**
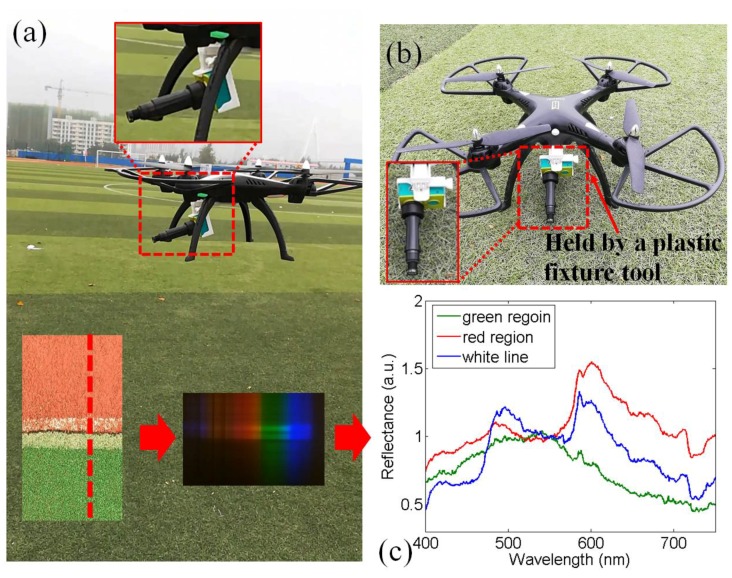
The compact imaging spectrometer can be installed in a consumer UAV for spectral detection. (**a**,**b**) show two views of the aerial spectral detection system, respectively. We flew the system over an athletic field to measure reflectance from the field and the track. The reflectance of the green artificial grass, the red plastic track, and a white line in the track are shown. (**c**) Spectra of three detected objects.

UAVs are widely used both for scientific and consumer purposes. Recently, scientists have proposed using UAV to achieve remote sensing with high spatial and spectral accuracy [21]. In this section, we demonstrate that our compact imaging spectrometer can be installed on a consumer UAV to perform remote detection. We bought a UAV (~50 dollars) from an Original design manufacture (OEM) factory. Figure 5 shows that the spectrometer can be easily installed on this UAV. This UAV could take the imaging spectrometer into the air. Our compact imaging spectrometer was installed on the UAV via a plastic fixture tool, as shown in Figure 5b. This experiment was performed in a football field. While the imaging spectrometer was in the air, a spectral image was taken (the inset of Figure 5a) that contained the reflectance from the plastic track and the artificial grass. The spectra of the artificial grass (green region), plastic track (red region), and the white line are shown in Figure 5c. This experiment shows that our imaging spectrometer can enable airborne remote sensing. The imaging spectrometer would apply addition torque to the UAV. Therefore, the UAV with imaging spectrometer struggles to maintain a steady flight path (a better UAV with motorized selfie stick can solve this problem).

## 4. Conclusions

In this work, we designed a compact imaging spectrometer (35 g in weight, 18 mm in diameter, and 72 mm in length) for remote sensing. Instead of incorporating it with professional equipment of optics and fine mechanics, we combined the compact imaging spectrometer with consumer electronics products, such as a selfie stick, a waterproof dome port, and a consumer UAV. We can share the detailed information of optical elements via email. With our system, we can acquire a hyperspectral data cube of environmental objects and human faces and obtain the reflectance spectra of chlorophyll. The raw data and Matlab code are available from the authors upon request via email. In the future, we will utilize a selfie stick with a better gyroscope module and inertial measurement unit (IMU) system to restrain the vibration caused by wind. Furthermore, this imaging spectrometer can be easily installed on both a waterproof dome port and a UAV for underwater or airborne spectral detection. This compact imaging spectrometer opens up the possibility for on-site and distributed multispectral imaging for various application fields.

In addition, an imaging spectrometer is an important module for various useful instruments. For example, light sheet microscopy [22,23,24], which is a rapid three-dimensional imaging system, can be improved to a four-dimensional imaging system by replacing the CMOS camera with an imaging spectrometer [25]. Furthermore, with minor changes, an imaging spectrometer can be updated to a continuous-wave laser lidar [26], which can be utilized in underwater Raman imaging and oil leak detection [27,28]. The above works have shown that the combination of an imaging spectrometer module with other advanced optical systems can lead to novel instruments. Therefore, we believe that the utilization of compact imaging spectrometers has the potential to yield new applications in the future.
